# Antitumor Effect of *Inula viscosa* Extracts on DMBA-Induced Skin Carcinoma Are Mediated by Proteasome Inhibition

**DOI:** 10.1155/2021/6687589

**Published:** 2021-03-27

**Authors:** Ouadie Mohamed El Yaagoubi, Ayoub Lahmadi, Abdelhakim Bouyahya, Hassan Filali, Hamid Samaki, Said El Antri, Souad Aboudkhil

**Affiliations:** ^1^Laboratory of Biochemistry, Environment and Agri-Food (URAC 36), Faculty of Sciences and Techniques-Mohammedia, Hassan II University, Casablanca, Morocco; ^2^Laboratory of Human Pathologies Biology, Department of Biology, Faculty of Sciences, Mohammed V University, Rabat, Morocco; ^3^Genomic Center of Human Pathologies, Faculty of Medicine and Pharmacy, Mohammed V University, Rabat, Morocco; ^4^National Institute of Social Action (INAS), Tangier, Morocco

## Abstract

The aim of this work is to evaluate the antitumor effect mediated by the proteasome inhibitors of *Inula viscosa* extracts on skin carcinogenesis. Female Swiss albino mice were divided into five groups depending on the combination of skin cancer-inducing 7,12-dimethylbenz(a)anthracene (DMBA) and extract of *Inula viscosa* treatments. Histology of the affected skin and measurement of proteasome activity were performed to demonstrate the effect of *Inula viscosa* on mice. The identification of the molecules responsible for this inhibitory activity was carried out through the docking studies. The results showed that *Inula viscosa* extracts inhibit the development of papilloma in mice. Therefore, the best chemopreventive action of *Inula viscosa* was observed on mice in which extract treatment was performed before and after the induction of skin carcinogenesis. It was revealed that the ingestion of extracts *Inula viscosa* delays the formation of skin papillomas in animals and simultaneously decreases the size and number of papillomas, which is also reflected on the skin histology of the mice treated. Structure–activity relationship information obtained from component of *Inula viscosa* particularly tomentosin, inuviscolide, and isocosticacid demonstrated that distinct bonding modes in *β*_1_, *β*_2_, and *β*_5_ subunits determine its selectivity and potent inhibition for *β*_5_ subunit.

## 1. Introduction

The ubiquitin–proteasome system (UPS) plays a fundamental role in intracellular proteolysis. Furthermore, its central role is the degradation of abnormal proteins. Indeed, it is vital for the regulated degradation of intracellular proteins and is directly involved in the regulation of most biological processes such as cell cycle, apoptosis, muscle differentiation, or immune response [1]. Recently, proteasome levels have been suggested as a biomarker of various cancer diseases [2–4].

At the heart of this system is the proteasome, which is an essential protease in eukaryotes. The proteolytic core of this complex called proteasome 20S is shaped like a hollow cylinder containing the catalytic sites in internal cavity. Crystal structure (PDB: 4R3O) shows that the active sites of the 20S proteasome are mainly located on the *β*_1_, *β*_2_, and *β*_5_ subunits, which have caspase-like (C-L), trypsin-like (T-L), and chymotrypsin-like (ChT-L) activities, respectively.

Recent studies on the function of these subunits demonstrated that inhibition of *β*_5_ subunit could achieve therapeutic effects [5–7]. The proteasome inhibition for anticancer therapy, particularly by natural compounds, has produced distinct and promising outcomes of cancer treatment [8, 9]. Therefore, the use of medicinal plants is an ancient practice because these plants have generated very encouraging therapeutic results with fewer side effects observed during their use since they are less offensive and less harmful to the body [10–12]. Besides, they are relatively less expensive than modern medicine. Given this explosion in the use of medicinal plants and their beneficial effects, we thought it would be interesting to examine the antitumor effect of *Inula viscosa* on the progression of skin cancer in mice.

*Inula viscosa* (L.) Aiton (Compositae) locally called “Magramane” is a perennial herbaceous plant distributed in different regions of the Mediterranean Basin [13]. Therefore, the plant has been used in traditional medicine for treatment of different diseases, including anti-inflammatory [14], anthelmintic [15], antipyretic, antiseptic, and antiphlogistic activities [16, 17]. In fact, crude extracts prepared from different parts of *Inula viscosa* demonstrated antioxidant [18] and cytotoxic activities on large variety of cancerous cells [17]. The present study is aimed at investigating the antitumor promoting effect and proteasome inhibitors of *Inula viscosa* extract in a specific model of DMBA/croton oil-induced skin carcinoma in mice. Indeed, the study of structure–activity relationships of bioactive molecules of *Inula viscosa* extract allows us to visualize its powerful inhibition for the subunits of the proteasome.

## 2. Materials and Methods

### 2.1. Preparation of Extracts from *Inula viscosa*

*Inula viscosa* L. was collected from the regions of Taza, Morocco. Thereafter, it was dried away from light and humidity at room temperature. Once dried, the plant material was reduced to powder using electric propeller mill and extract under reflux. The method used is called maceration. Ethanol extraction was performed at the ratio of 10% (*w*/*v*) for 3 h under agitation [19]. The separation of the ethanol from the extract of *Inula viscosa* was carried out using a rotary evaporator at a temperature of 33°C and at 78 rpm. Once obtained, the extract is stored at 4°C in the dark until use.

### 2.2. Animals

Female Swiss albino mice, 8–10 weeks old of age and weighing (25 ± 30 g), were obtained from the pet shop of our institute and were housed in polypropylene cages. The animals were acclimatized for 1 week before starting the experiment. Mice were fed with commercially available food pellets and tap water ad libitum and maintained on standard housing conditions under a controlled atmosphere with 12 : 12 h light/dark cycles with ambient temperature of 25 ± 5°C, and humidity at 50 ± 10%. Animal handling and experimental protocol were conducted according to the guidelines of the Institutional Ethical Committee (IEC).

### 2.3. Experimental Groups of Mice

In order to determine the nonlethal dose of the *Inula viscosa* plant extract, a toxicity test was performed. In fact, decreasing concentrations and/or volumes of the extract were used until obtaining a dose that does not cause lethality on mice. This dose was then used in the same mouse for 4 days, a period chosen to mimic the protocol used during the treatment with the extracts. The volume used was 25 *μ*L and adjusted to 100 *μ*L using phosphate-buffered saline (PBS). The anticancer potential of the prepared extract was evaluated by administering 100 *μ*L of the extract to mice as skin cancer was in parallel induced.

To induce carcinogenesis in mice, approximately 2.5 cm^2^ of dorsal body wall hair was removed by shaving with electric clippers followed by the application of depilatory cream one day prior to the beginning of the experiment. Thereafter, the mice were initiated by a single topical application of DMBA (100 *μ*g/100 *μ*L acetone) on shaved dorsal skin (initiation phase) and, 2 weeks later, promoted by twice-weekly topically applications of 1% croton oil (1 *μ*L/100 *μ*L acetone) at the same site for 20 weeks (promotion phase). The normal control mice received only acetone in place of DMBA and croton oil [20]. Animals were divided into five groups (10 mice/group) as follows: Group 1: treated by intraperitoneal injection of *Inula viscosa* extract during the initiation phase; Group 2: treated by intraperitoneal injection of *Inula viscosa* extract during the promotion phase; Group 3: treated by intraperitoneal injection of *Inula viscosa* extract during the initiation and promotion phase; Group 4: treated by intraperitoneal injection of PBS (tumor control); and Group 5: treated by intraperitoneal injection of PBS (normal control).

The mice in group 1 were treated with intraperitoneal injection of 100 *μ*L of the prepared extracts, one day before the initiation phase, 30 min before initiation, and one day after the initiation phase.

The mice in group 2 were treated with intraperitoneal injection of 100 *μ*L of the prepared extracts, one day before the promotion phase, 30 min before the promotion, and one day after the promotion phase.

The mice of group 3 were treated with intraperitoneal injection of 100 *μ*L of the prepared extracts, one day before the initiation phase, 30 min before the initiation, and one day after the initiation phase.

Mice in the group 4 (tumor control) and group 5 (normal control) were treated with 100 *μ*L of PBS and around the days of DMBA and croton oil application with intraperitoneal injections carried out in the same way as in the mice treated with the extracts.

### 2.4. Morphologic Observation of Papilloma Development

During the 20 weeks of experiments, mice were observed daily for the appearance of skin papillomas tumor, volume, and body weights, which were recorded at interval of 7 days until sacrifice. Tumors were counted, measured, and scored weekly as clinically apparent papillomas (typically well-demarcated, asymmetrical, pedunculated, or dome-shaped papules, without erosion or ulceration) or clinically apparent carcinomas (poorly demarcated, asymmetrical, sessile, or dome-shaped papules with erosion or ulceration). Tumors were evaluated by visual inspection to the experimental groups.

### 2.5. Histological Evaluation

Collected skins (tumors and normal skin) were fixed in 10% neutral formalin for 24 h and passed through ascending grades of ethyl alcohol starting from 60% to 95%. The dehydrated tissues were then soaked in toluene and then transferred to molten paraffin (60°C), which was poured into metal molds, and the tissues were set accordingly. Serial microtome sections in the form of paraffin ribbon were made at a thickness of 4 *μ*m. The tissues were then floated in a tray covering lukewarm water (58°C) to stretch the tissues. Tissue sections (3–4 *μ*m) were taken for hematoxylin–eosin (HE) staining and histopathological evaluation [21].

### 2.6. Cell Lysis

The serum is recovered from a mouse blood sample and gradually cooled to 4°C and -20°C. Lymphocyte the pellet is recovered by lysis of red blood cells. First, we proceed to the preparation of the white cell pellet. Briefly, red cells of the blood samples were broken by adding a solution of Tris-EDTA (20/5) to whole blood after centrifugation, the supernatant is removed, and this procedure was repeated until a clear cell layer is obtained. Thereafter, 200 mU/L of lysis buffer (10 mM KCl, 10 mM NaCl, 10 mM HEPES, 1 mM EDTA pH 7.1, 0.1 mM DTT, and 1% Triton, supplemented with protease inhibitors (PMSF 2 nM)) was added on each sample. The cells are disrupted with alternating periods of 20 s break with 30 s rest to avoid excessive heating of samples that can cause denaturation of enzymes [22]. The protocol is repeated 3 times. The extracts obtained may be stored at -20°C until their use.

### 2.7. Measurement of Proteasome Activity

The catalytic activity (chymotrypsin-like) of the 20S proteasome on the fluorogenic peptide Leu-Leu-succinyl-Val-Tyr-Amido-4-methylcoumarin (Suc-LLVY-AMC) was determined after substrate incubation with the sample at 37°C for over 3 h. The fluorescence emitted after cleavage of the AMC is determined using a Fluorometer (Hoefer Scientific Instruments). The excitement of peptides, coupled at the AMC, is done through a 360/40 nm filter at a wavelength between 340 nm and 380 nm with a maximum of 360 nm. The fluorescence emitted after digestion of peptides coupled to the AMC reads on the 460/40 nm filter. To convert the fluorescence unit (FU) issued *μ*mol of used fluorophores, a standard AMC range was established from a stock solution of AMC at 10-3 m. The excitement of the AMC is done at a wavelength of 360/40 nm, and the signal is then recovered on the 460/40 nm filters [22].

### 2.8. Docking Studies

For this study, plant compounds were collected from the PubChem database. A total of 14 molecules are reported to be isolated from *Inula viscosa* L.; these molecules were considered for the study of molecular docking. Therefore, the X-ray crystalline 3D structure of the proteasome 20S (PDB: 4R3O, resolution 2.60 Å) was obtained from the Data Bank Protein [23] and prepared by Discovery Studio 2020 [24] by removing water molecules, and polar hydrogen was added, the 4r3o PDB file containing 28 chains, 2 chains (1,M) for *β*_1_, 2 chains (K,Y) for *β*_2_, and 2 chains (L,Z) for *β*_5_. The rest of the chains are removed and only the *β*_1_, *β*_2_, and *β*_5_ chains kept to speed up and simplify the calculations.

The three molecules selected according to the virtual screening were used to perform molecular docking against the target PDB (4R3O). The AutoDock Vina [25] and MGLTools programs [26], were used with default settings. Carfilzomib was considered a positive control.

### 2.9. Virtual Screening

High-speed virtual screening was performed using the iGEMDOCK (Generic Evolution Method for Docking) program [27]. The *in silico* screening of 14 compound extracted from *Inula viscosa* was performed using the PDB code of the targets (PDB ID: 4R3O); the screening score, which is based on total energy calculations (total energy = van der Waals (VdW) + hydrogen bond (HBond) + electrostatic), was calculated using iGEMDOCK v2.1.11.

The standard parameters used for screening, population size, generations, and number of solutions, were set at 300, 70, and 2, respectively. Energy-based results were analyzed, and 9 potential inhibitors were selected based on stability for further detailed analyses.

### 2.10. Visualization and Analysis of Results

The results were visualized by the Discovery Studio Visualizer [28] and PyMOL [30]. The results of the molecules showed an interesting docking score, and their positioning inside the active site was also compared as well as type of interactions established by each molecule inside the active site.

### 2.11. Prediction of ADMET

To develop a drug, several steps are necessary, starting with target identification and ending with ADMET prediction. Therefore, the early determination of these properties is very necessary to reduce the cost and also the time of the drug discovery process. These parameters include the absorption of the drug (absorption), the distribution in the body (distribution), the biochemical remodeling (metabolism), excretion, and toxicity. In this perspective, the best three molecules selected on the basis of their score energy and which showed better affinity have been evaluated to determine these pharmacokinetic parameters *in silico*, to prevent the failure of these compounds in clinical trials and increase their chances of reaching the stage of drug candidates in the future [31, 32].

### 2.12. Statistical Data Analysis

All data obtained are represented as the mean ± standard error of the mean (SEM). The results were computed statistically (IBM SPSS statistics 20 Software Package) using one-way ANOVA. In all tests, the level of statistical significance was set at *p* < 0.05.

## 3. Results and Discussion

### 3.1. Skin Papillomas Assessments

**Figure 1:**
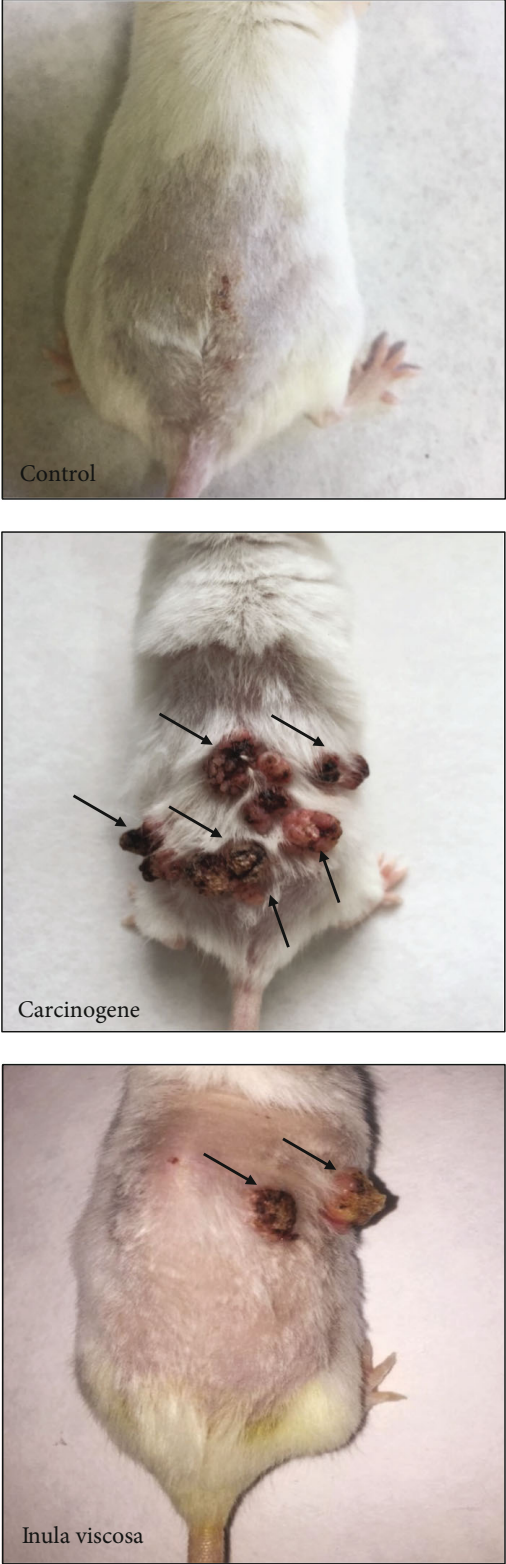
Representative graphs of the effect of *Inula viscosa* L. extract on the evolution of skin carcinoma on Swiss albino mice.

In order to evaluate the antitumor potential of *Inula viscosa* extract, three groups of mice were used, including a control group, a carcinogenesis group, and a group treated with *Inula viscosa* extract during the tumor initiation and promotion phases (Figure 1). The skin of the control mice did not develop any papilloma growth, whereas all mice in the carcinogenesis group demonstrated increasing formation of papillomas. Therefore, the mice treated with intraperitoneal injections of *Inula viscosa* extract demonstrated a reduced occurrence of the growth of papilloma compared to the carcinogen mice. Moreover, the best chemopreventive action of *Inula viscosa* was observed in mice in which extract treatment was performed before and after the induction of skin carcinogenesis.

### 3.2. Effect of the *Inula viscosa* during the Initiation Phase

**Figure 2:**
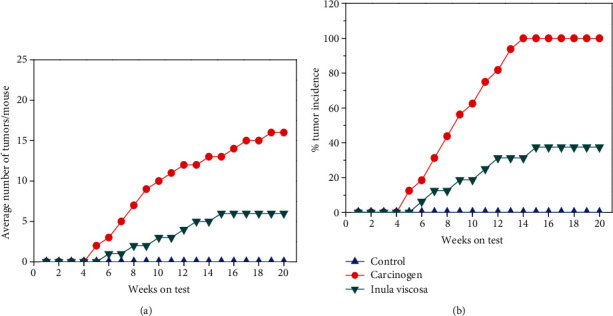
The treatment with plant extract is applied only during the initiation phase. (a) Tumor multiplicity (average number of papillomas per mouse). (b) Tumor incidence (percentage of mice with papillomas).

The carcinogenic mice (Figure 2(a)) not treated with plant extract showed a mean number of papillomas of 16 ± 2.15 after 20 weeks. However, a significant reduction of papillomas was observed in the group treated with *Inula viscosa* (6 ± 1.5). Therefore, the control mice did not produce any papilloma on their skin but 100% of the DMBA-treated mice developed papilloma growth until the end of 20 weeks (Figure 2(b)). *Inula viscosa*-treated groups of mice demonstrated slower growth of papillomas (37.5%).

In this study, we found that appearance of papillomas depends on the type of treatment. Indeed, it was noticed that the appearance of the tumors increases in number and size with time. On the other hand, for the control mice, no lesions or tissue damage were observed, and the appearance of papillomas is very similar to the control mice.

### 3.3. Effect of the *Inula viscosa* during the Promotion Phase

**Figure 3:**
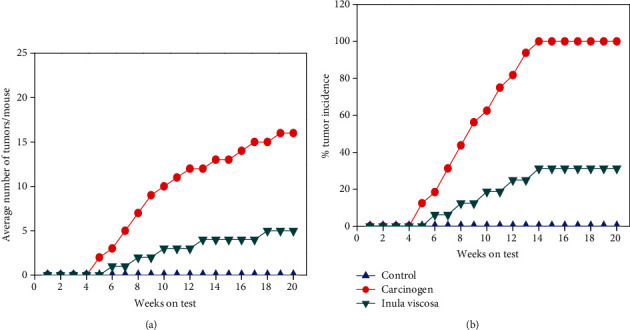
The treatment with plant extract is applied only during the promotion phase. (a) Average number of papillomas per mouse. (b) Tumor incidence (percentage of mice with papillomas).

To evaluate the effect of *Inula viscosa* extract on tumor development in mice, abdominal injections were applied during the promotion phase. The carcinogenic mice (Figure 3(a)) not treated with plant extract showed average number of papillomas of 16 ± 2.15 after 20 weeks. Furthermore, a significant reduction of papillomas was observed in the group treated with *Inula viscosa* (5 ± 1.25). Therefore, the control mice did not produce any papilloma on their skin but 100% of the DMBA-treated mice developed papilloma growth until the end of 20 weeks. *Inula viscosa*-treated groups of mice (Figure 3(b)) demonstrated slower growth of papillomas (31.25%).

### 3.4. Effect of the *Inula viscosa* during the Initiation and Promotion Phase

**Figure 4:**
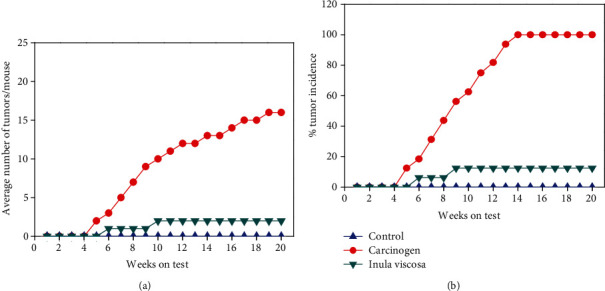
The treatment with plant extract is applied during the promotion and initiation phase. (a) Average number of papillomas per mouse. (b) Tumor incidence (percentage of mice with papillomas).

The treatment of carcinogenic mice with *Inula viscosa* extract during the initiation and promotion phase showed a reduction of the tumor load (number of papillomas/mice); the mice stabilized in 2 papillomas whereas the number of papillomas in carcinogenic mice were 16 ± 2.15 (Figure 4(a)) with inhibition growth of papillomas, which can be as much as 87.5%. (Figure 4(b)) after 20 weeks of treatment. Therefore, the results obtained clearly show the presence of a protective effect due to the extract treatment. Our results are in agreement with some studies [33, 34]. Phytotherapy has proved to be very promising. Indeed, *in vivo* study showed that treatment with gallic acid induces the inhibition of cancer cell proliferation, through the suppression of DMBA action and croton oil by modulating the antioxidant and MMP-2/MMP-9 in Swiss albino mice [32]. In contrast, the results obtained suggest that extracts from *Inula viscosa* have a strong antitumor effect effective in preventing DMBA-induced skin cancer. Hence, this observed difference could be explained by the fact that the process of carcinogenesis is multifactorial and made up of several steps. Therefore, for a good control, the treatment should be applied before, during, and after the installation of cancer.

DMBA is a very potent carcinogen, which can lead to mutations in cellular DNA. The critical event in the initiation of carcinogenesis by DMBA is the conversion of DMBA to reactive oxygen species by a set of enzymes that metabolize xenobiotics. Croton oil is rich in phorbol esters; it can act as tumor promoters by activating protein kinase C. The increase in the concentration of active oxygen, organic peroxides, and radicals in cells can also promote neoplastic growth of initiated cells [34]. The key mechanism of action of the majority of chemopreventive agents is their potential to prevent either the metabolic activation of procarcinogens or the neutralization of reactive metabolites and free radicals generated during the process of carcinogenesis [35]. In cancer cells, chemopreventive agents exert cytotoxicity in cancer cells and target different stages of cancer progression by inhibiting angiogenesis, preventing metastasis, and inducing apoptosis [36]. The ability of *Inula viscosa* bioactive molecules to selectively activate apoptotic cascades in cancer cells, inhibit the activation of carcinogens, and prevent further promotion could be the explanation for its demonstrated antitumor effects.

### 3.5. Histopathological Findings

**Figure 5:**
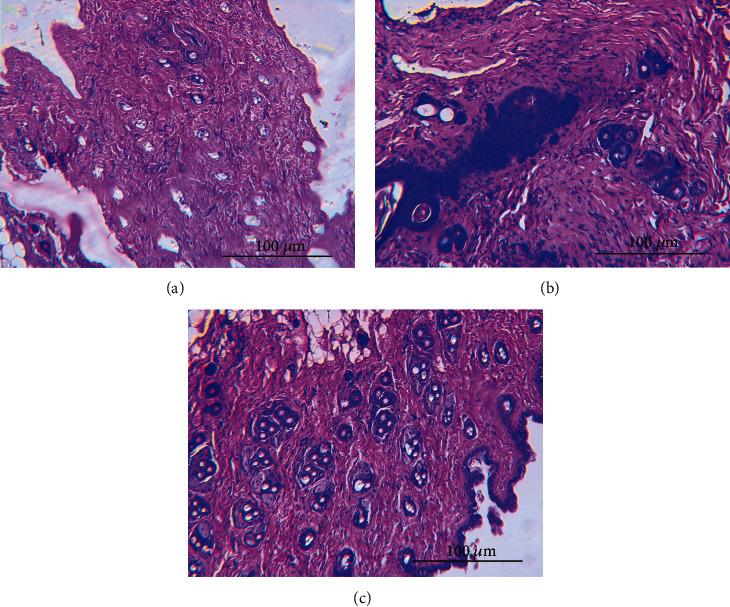
Hematoxylin and eosin staining of cross-sections of mouse skin and observed under light microscope. (a) Skin of control mice treated with the vehicle shows a normal distribution of the deferent skin layer. (b) Carcinogenesis group (DMBA/croton oil) showed epithelial vascular lesion, epidermal hyperplasia with hyperkeratosis, tumor nest, parakeratosis, spongiosis, and marked acanthosis. (c) Mouse skin treated with *Inula viscosa* extract showed normal skin structure and architecture with mild to moderate prevention of skin carcinogenesis.

Skin is made of three large layers: epidermis, dermis, and hypodermis. This is made up of a variety of distinct cell types, the primary components being keratinocytes and melanocytes. Keratinocytes, which compose 95% of the epidermis, are organized into four layers. The inner layer is the stratum germinativum (stratum basale, basal layer), from which column-shaped keratinocytes divide to migrate to the next layer. Two layers of small, undifferentiated epidermis cells differentiate into different layers following exposure to a local carcinogen. The papillary layer in the dermis is a subepithelial layer of tightly arranged, fine collagenous fiber and fiber bundles. The dermis reticular layer comprises coarse collagenous fibers of considerable thickness [37]. Those are the characteristics control mice that showed a normal distribution of different layers of the skin: no hyperplasia and no cell proliferation was observed (Figure 5(a)). In contrast, analysis of histological sections of mice developing tumors (Figure 5(b)) showed a typical change in the skin illustrated by thickening of the epidermis, epidermal cells invasion into the dermis, hyperkeratosis, and acanthosis of the epidermis. Therefore, histological sections of mice treated with the *Inula viscosa* plant extract (Figure 5(c)) showed normal structure and architecture of the skin with mild to moderate prevention of skin carcinogenesis.

Nonetheless, according to these data, it can be proposed that our extract exerts its antitumor effects either at the initiation phase by protecting the DNA from mutations caused by the action of DMBA [32] or during the promotion phase through the neutralization of the microenvironment of free radicals [33], which favor the setting up of a carcinogenesis process. The results obtained showed that our extract exerts their effect during both phases. This translates into the best reduction in the number of papillomas observed when the mice are treated during both phases (initiation and promotion) compared to those treated during the initiation phase alone, and/or the promotion phase alone.

### 3.6. Measurement of Chymotrypsin-Like Proteasomal Activity

**Figure 6:**
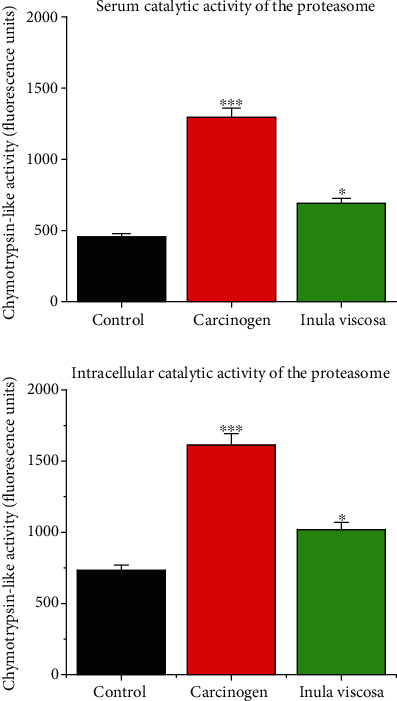
Chymotrypsin-like catalytic activity in intracellular and serum levels. The difference is statistically significant (∗*p* ≤ 0.05; ∗∗*p* ≤ 0.01; ∗∗∗*p* ≤ 0.001).

It can be seen from Figure 6 that at the serum level, the catalytic activity of the proteasome of mice from groups treated with *Inula viscosa* extracts (691.14 ± 8.55 FU) was significantly lower (*p* ≤ 0.001) compared to that measured in mice from the carcinogenesis group (1294 ± 34.1 FU), whereas the lowest catalytic activity was measured in the control group (455.63 ± 22.75 FU). The intracellular proteasome catalytic activity of mice in *Inula viscosa*-treated groups (1020 ± 18.1 FU) was lower compared to that measured in the carcinogenesis group (1612 ± 30.2 FU), while mice in the control group still showed the lowest proteasome catalytic activity (733 ± 36.65 FU).

Therefore, the catalytic activity of proteasome at serum and intracellular levels was very important in mice of the carcinogenic group than in mice of the groups treated with *Inula viscosa* extract. These data are in agreement with several findings using the approach of proteasome inhibition by chemically synthesized inhibitors [38–40]. Indeed, Lavabre et al. in 2001, in a study of solid tumors, showed a total regression of the tumors accompanied by a decrease in the concentration of proteasome as well as its catalytic activity with treatment with chemically synthesized proteasome inhibitors such as epoxomicin and bortezomib. Furthermore, subsequent studies performed in our laboratory by Filali et al. [22] on Moroccan patients with hematological malignancies suggest that treatment with proteasome inhibitors, in addition to tumor regression, induces a decrease in proteasome concentration and catalytic activity. Hence, it can be considered that proteasome could be a new factor in the diagnosis and follow-up of treatment in cancer diseases [41, 42]. Based on these data, the proteasome could be a key factor in the neoplastic differentiation of cells, a novel cancer biomarker, and a key element in the clinical follow-up of patients with skin cancer. Hence, the analysis of the catalytic activity provides information on the functionality of the complex and consequently on its molecular nature at the serum and subcellular levels. Several studies [42, 43] demonstrate that only the inhibition of chymotrypsin-like activity, carried by the *β*5 subunits, is sufficient to allow a significant reduction in the rate of protein degradation while the inactivation of the other sites, trypsin-like and PGPH, has little effect on total proteolysis.

Treatments based on the *Inula viscosa* extract have been shown to be effective in the treatment of skin carcinoma. In addition, this extract contains phenolic compounds and/or flavonoids known for their antioxidant effects [44, 45] and contains bioactive molecules with structures similar and close to those of chemically synthesized proteasome inhibitors, capable of effectively inhibiting the ubiquitin proteasome complex, more specifically the catalytic activity of the latter. Based on these data, it can be concluded that treatment with *Inula viscosa* extract causes a regression of the tumor load with a decrease in serum and intracellular activities.

Consequently, the treatment with the plant extract of *Inula viscosa* revealed significant results and could constitute therefore a new approach for the treatment of skin carcinoma.

### 3.7. Virtual Screening

The analysis of the type of binding and the calculation of the energy score of 14 compounds extracted from *Inula viscosa* with the proteasome 20S (PDB: 4R3O) allows the selection of the best compounds. Three molecules were selected on the basis of their low energy values. The energy values of tomentosin, inuviscolide, and isocosticacid are -65.53, -64.53, and -60.68, respectively, for *β*_1_, and -62.63, -62.09, and -57.90 for *β*_2_, and -61.23, 62.41, and 60.37 for *β*_5_ as shown in (Table 1).

### 3.8. Molecular Docking

The three molecules obtained after the high throughput screening were used for further study using molecular docking to study their stability and interactions with the *β*_1_, *β*_2_, and *β*_5_. Carfilzomib was considered a positive control.

The binding energy of the selected candidates ranged from -6.3 to -6.1 kcal/mol for *β*_1_ and from -6.5 to -6 kcal/mol for *β*_2_, and from 6.2 kcal/mol to 6 kcal/mol for *β*_5_, with all three molecules having higher energy than carfilzomib (-5.2 kcal/mol) for the three targets, as shown in (Table 2). The resulting molecule, all three compounds, had a greater affinity with the active site of *β*_1_, *β*_2_, and *β*_5_.

### 3.9. Visualization and Analysis of Results

**Figure 7:**
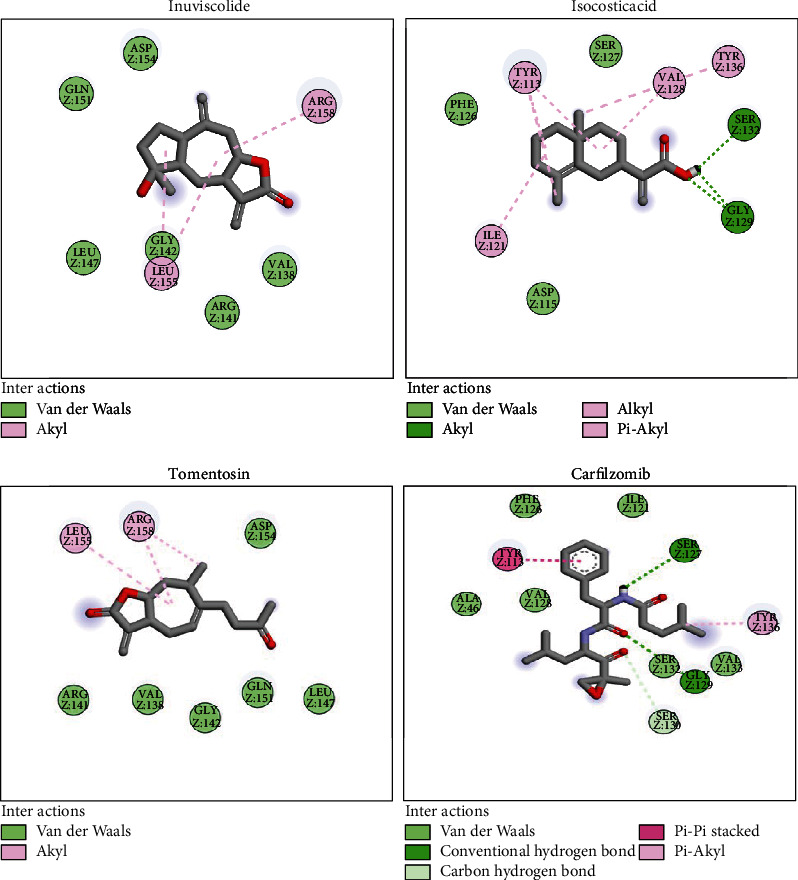
Different types of interactions between tomentosin, inuviscolide, isocosticacid, and carfilzomib with 20S proteasome *β*_5_ subunit.

We visualized the positioning of these three molecules with *β*_5_ (Figure 7); also, the one of carfilzomib shows the docking results and the types of interactions with the amino acids within the active site.

The molecular docking results are shown in (Figure 7) which indicate that tomentosin, inuviscolide, isocosticacid, and carfilzomib compounds are stabilized in the pocket of the *β*_5_ receptor by various interactions. The three compounds that are the most active molecules are stabilized by hydrogen-bonding interactions, pi-alkyl interactions, carbon hydrogen, pi-sigma carbon bonds, and Van der Waals interactions (Table 3).

### 3.10. ADMET Properties

The pharmacokinetic properties of three compound have been determined; the compounds are adsorbed when they fulfil the Lipinski rule, where a good drug should have a LogP partition coefficient of less than 5, a weight (MW) of less than 500 Da, number of HBA < 10, number of HBD < 5, and rotational bonds of less than 10 [46] (Table 4).

The objective of the ADMET preclinical study is to eliminate weak candidates and focus on successful drug candidates. This study addresses the applicability of the three compounds proposed as anticancer agents using the virtual properties, absorption, distribution, metabolism, excretion, and toxicity, which are crucial actors in drug development. The pharmacokinetic properties have been calculated using admetSAR. Therefore, penetration of the blood-brain barrier (BBB), human intestinal absorption (HIA), Caco-2 cell permeability, and AMES test are used to specify drug-like properties. Penetration of the blood-brain barrier (BBB) is an important property since it determines whether or not drugs can cross the blood-brain barrier and also exert its effect on the brain [47].

Compounds with a BBB less than 1 are considered poorly distributed in the brain. The BBB permeability results in (Table 5) show a nonpenetrating BBB for novel anticancer compounds. The molecule with less than 30% absorbance is considered poorly absorbed, indicating that all compounds tested could be absorbed from the human gut; however, the cytochrome P450 subtypes CYP2D6 and CYP3A4 indicate that the compounds inuviscolide and tomentosin could not be substrates or inhibitors for the two major subtypes and therefore would likely not be metabolized. Fortunately, our best candidate compound isocosticacid showed no acute toxicity and no mutagenic effects compared to the Ames test data (Table 5).

## 4. Conclusions and Perspectives

In conclusion, this study revealed that the treatment with *Inula viscosa* extract shows a strong inhibition for the appearance of tumors in the form of papillomas, which is clearly demonstrated by the decreased activity at the serum and intracellular levels. Therefore, the docking studies remain a very effective tool and essential study to identify the degrees of structural similarity and the percentage of predictive inhibition of proteasome subunits by bioactive molecules. The data obtained clearly show that *Inula viscosa* extract contains bioactive molecules with a much greater proteasome inhibition potentials than the chemically synthesized inhibitors and could be better new candidates in targeted therapy against skin carcinoma. However, it would be interesting to isolate and purify these molecules particularly (tomentosin, inuviscolide, and isocosticacid), in order to test them, in a later work, on other cancerous diseases.

## Figures and Tables

**Table 1:** Screening results of 14 compounds extracted from *Inula viscosa* with 3D structures of proteasome *β*_1_, *β*_2_, and *β*_5_.

Compound name	Total binding energy (kcal mol^−1^)			Structure
	*β*_1_	*β*_2_	*β*_5_	
Tomentosin	-65.53	-62.63	-61.23	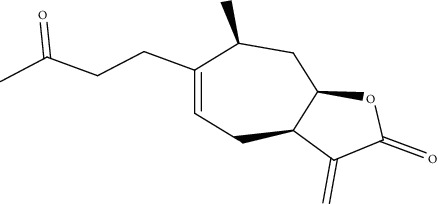
Inuviscolide	-64.53	-62.09	-62.41	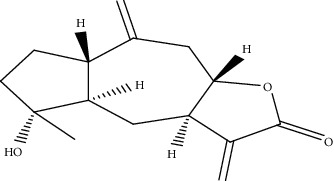
Isocosticacid	-60.68	-57.9	-60.37	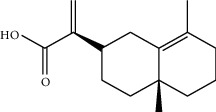
Carfilzomib	-86.07	-91.79	-99.58	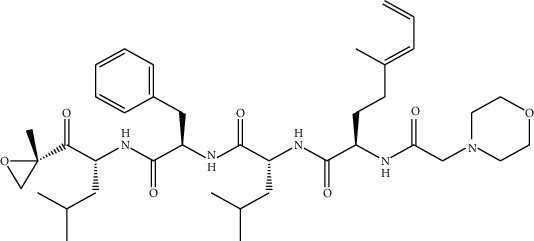

**Table 2:** Stowage results showing the best conformation, ranked according to their binding affinity.

Compound name	Total binding energy (kcal mol^−1^)		
	*β*_1_	*β*_2_	*β*_5_
Tomentosin	-6.3	-6.5	-6.2
Inuviscolide	-6.2	-6.4	-6.1
Isocosticacid	-6.1	-6	-6
Carfilzomib	-5.1	-5	-5.2

**Table 3:** Interactions of the proposed compounds with the *β*_5_ receptor.

Compounds	Residues
Tomentosin	Arg(70),Leu(66),Asn(63),Tyr(67),Phe(83),Leu(75),Val(64),Ala(79)
Inuviscolide	Glu(151),Asp(154),Leu(147),Leu(155),Gly(142),Arg(141),Val(138),Arg(154)
Isocosticacid	Glu(117),Asp(115),Tyr(113),Tyr(136),Ile(121),Phe(126),Val(128),Ser(127),Gly(129)
Carfilzomib	Gly(47),Gly(98),Ala(46),Val(128),Tyr(113),Ile(121),Phe(126),Ser(132),Ser(127,Gly(129),Tyr(136),Val(133)

**Table 4:** Molecular properties for predicting the drug sensitivity of 14 compounds extracted from *Inula viscosa.*

Compound	MW	LogP	HB acceptor	HB donor	Rotating bonds
Inuviscolide	248.32	2.2114	3	1	0
Isocosticacid	234.33	3.934	2	1	2
Tomentosin	248.32	2.8097	3	0	3
Carfilzomib	719.91	2.5835	8	4	24

**Table 5:** Pharmacokinetics and toxicity evaluation of compounds from *Inula viscosa*.

	Inuviscolide	Isocosticacid	Tomentosin	Carfilzomib
*Absorption and distribution*				
Blood-brain barrier (LogBB)	0.018	0.341	0.14	-1.082
Intestinal absorption (human)	97.306	93.881	97.922	55.371
Caco-2 permeability	1.288	1.593	1.343	0.056
*Metabolism*				
Substrate CYP2D6	No	No	No	No
Substrate CYP3A4	No	Yes	No	Yes
Inhibitor CYP1A2	No	No	No	No
Inhibitor CYP2C9	No	No	No	No
Inhibitor CYP2D6	No	No	No	No
Inhibitor CYP2C19	No	No	No	No
Inhibitor CYP3A4	No	No	No	Yes
*Excretion and toxicity*				
Clearance	1.057	1.162	1.276	1.027
AMES toxicity	No	No	No	No
Carcinogens	No	No	No	No

## Data Availability

All used data in this study were cited in the manuscript.

## Additional Points

The proteasome inhibition for anticancer therapy, particularly by natural compounds, has produced distinct and promising outcomes of cancer treatment. The use of a proteasome inhibitor might be an effective strategy in the treatment of skin cancer. *Inula viscosa* (L.) Aiton (Compositae) is a medicinal plant distributed in different regions of the Mediterranean Basin. Therefore, the plant has been used in traditional medicine for treatment of different diseases, including cancer treatment. *In vivo* studies showed that *Inula viscosa* extracts inhibit the development of skin carcinoma induced by DMBA in the mouse. Docking studies showed that *Inula viscosa* extract contains bioactive molecules with a much greater proteasome inhibition potentials than the chemically synthesized inhibitors and could be better new candidates in targeted therapy against skin carcinoma.
